# The Elongation Complex Components BRD4 and MLLT3/AF9 Are Transcriptional Coactivators of Nuclear Retinoid Receptors

**DOI:** 10.1371/journal.pone.0064880

**Published:** 2013-06-10

**Authors:** Sébastien Flajollet, Christophe Rachez, Maheul Ploton, Céline Schulz, Rozenn Gallais, Raphaël Métivier, Michal Pawlak, Aymeric Leray, Al Amine Issulahi, Laurent Héliot, Bart Staels, Gilles Salbert, Philippe Lefebvre

**Affiliations:** 1 European Genomic Institute for Diabetes (EGID), FR 3508, Lille, France; 2 INSERM UMR1011, Lille, France; 3 Univ Lille 2, Lille, France; 4 Institut Pasteur de Lille, Lille, France; 5 Unité de Régulation Epigénétique, URA 2578 du CNRS, Département de Biologie du Développement, Institut Pasteur, Paris, France; 6 Interdisciplinary Research Institute, Univ Lille 1, USR 3078 CNRS, Biophotonique Cellulaire Fonctionnelle, Villeneuve d’Ascq, France; 7 Equipe SPARTE, UMR CNRS 6026-Université Rennes 1, Rennes, France; Baylor College of Medicine, United States of America

## Abstract

Nuclear all-trans retinoic acid receptors (RARs) initiate early transcriptional events which engage pluripotent cells to differentiate into specific lineages. RAR-controlled transactivation depends mostly on agonist-induced structural transitions in RAR C-terminus (AF-2), thus bridging coactivators or corepressors to chromatin, hence controlling preinitiation complex assembly. However, the contribution of other domains of RAR to its overall transcriptional activity remains poorly defined. A proteomic characterization of nuclear proteins interacting with RAR regions distinct from the AF-2 revealed unsuspected functional properties of the RAR N-terminus. Indeed, mass spectrometry fingerprinting identified the Bromodomain-containing protein 4 (BRD4) and ALL1-fused gene from chromosome 9 (AF9/MLLT3), known to associate with and regulates the activity of Positive Transcription Elongation Factor b (P-TEFb), as novel RAR coactivators. In addition to promoter sequences, RAR binds to genomic, transcribed regions of retinoid-regulated genes, in association with RNA polymerase II and as a function of P-TEFb activity. Knockdown of either AF9 or BRD4 expression affected differentially the neural differentiation of stem cell-like P19 cells. Clusters of retinoid-regulated genes were selectively dependent on BRD4 and/or AF9 expression, which correlated with RAR association to transcribed regions. Thus RAR establishes physical and functional links with components of the elongation complex, enabling the rapid retinoid-induced induction of genes required for neuronal differentiation. Our data thereby extends the previously known RAR interactome from classical transcriptional modulators to components of the elongation machinery, and unravel a functional role of RAR in transcriptional elongation.

## Introduction

Transcriptional activation by nuclear all-trans retinoic acid (atRA) receptors (RARs) stems from the concerted action of transcriptional coregulators whose role is to convert a repressive chromatin environment into an opened state, allowing the assembly of the transcription preinitiation complex (PIC). Chromatin opening and PIC assembly are the end result of ligand-induced conformational changes in the highly structured C-terminal activating function (AF)-2 domain of DNA-bound RARs, creating a protein-protein interaction interface that recognizes LXXLL-containing transcriptional coregulators. Distinct families of transcriptional coregulators are recruited to the RAR AF-2 in response to agonists, including the p160 family (SRC1, TIF2/GRIP1, AIB1/ACTR/pCIP), CBP/p300, which recruit or carry histone acetyltransferase activity, and the DRIP/TRAP/Mediator complex which controls the basal transcription initiation machinery [1].

The *Rarβ2* promoter is a paradigm for NR-mediated transactivation, and has provided considerable insights into RAR-controlled transcription. Detailed mechanistic studies using this promoter showed that RARα-driven transcription requires, in addition to the above mentioned transcriptional coregulators, protein complexes involved in DNA breakage and repair such as topoisomerase II, PARP-1 and PCNA [2]–[4] and appropriate post-translational modifications of corepressors [5]. Furthermore, histone H3 Serine10 (S10 H3) phosphorylation is concomitant to retinoid-induced *Rarβ2* activation [6]. This histone mark is known to favor the loading of the positive transcription elongation factor b (P-TEFb) on regulated promoters, which is further facilitated by BRD4/HUNK1, a bromodomain-containing transcription factor with high affinity for acetylated histones H3 and H4 and Mediator subunits [7]–[9]. Intriguingly, constitutively acetylated histones H3 and H4 reside at the *Rarβ2* promoter, favoring the permanent loading of RXR-RARα heterodimers onto the retinoic acid response element (RARE) located in this promoter [10]. In line with the possible involvement of P-TEFb in *Rarβ2* promoter activation process, the kinase subunit of P-TEFb CDK9 associates to this promoter in a ligand-controlled manner [11]. Thus a functional role of P-TEFb in retinoid-induced activation of the *Rarβ2* promoter can be hypothesized on the basis of this physical colocalization.

Beside the ligand-regulated AF-2 region that encompasses the ligand binding domain (LBD), RARs harbor other functional domains such as the DNA binding domain (DBD) and the poorly characterized, unstructured, ligand-independent N-terminal AF-1 domain. Little is known about the exact roles of RARα domains outside of the LBD in transcriptional regulatory processes. In addition to its recognized role in direct protein-DNA interaction, the DBD interacts with transcription factors such as RXRs, c-jun, BLZF1, NF-IL6, myb and TEL [12]. Similarly, RARα AF-1 engages into intra-molecular interactions with RARα AF-2 to activate transcription, according to a mechanism involving the recruitment of TFIIH subunits cyclin H to AF-2, and of the kinase cdk7 to AF-1 [1]. We have therefore further investigated this question by purifying putative RARα coregulators able to interact with RARα domains distinct from the AF-2 domain. Mass spectrometry fingerprinting confirmed that RARα AF-1 interacts with the p62 subunit of TFIIH. More strikingly, this approach revealed that the two mutually exclusive P-TEFb interactants AF9/MLLT3 and BRD4/HUNK1 [13], [14] bind to RARα in a ligand-independent manner, evidencing a physical connection between RAR and transcription elongation factors. AF9 and BRD4 played distinct roles in retinoid-induced transcription and neuronal differentiation as shown by microarray analysis of mRNAs from the mouse pluripotent cell line P19. We further show that RAR associates to transcribed regions of retinoid-regulated genes in an AF9 and BRD4-dependent manner, and as a function of P-TEFb activity.

## Materials and Methods

### Plasmids

Expression vectors for RAR and RXR, and reporter constructs have been described [15]. pSG5-RARα-ΔAF-1, and pcDNA3-AF-1 and pGFP-NLS-AF-1 were constructed by PCR amplification of the *Rar*α cDNA, followed by cloning in pSG5 (Stratagene, Santa Clara, CA), in pcDNA3 (Invitrogen, Carlsbad, CA) and in pEGFP-C1 (Clontech, Mountain View, CA) containing a nuclear translocation signal (NLS), respectively. cDNAs corresponding to the proteins identified by mass spectrometry were obtained as full length open reading frame clones from the Mammalian Gene Collection (N.I.H., Bethesda, MD) or from Origene (Rockville, MD). They were amplified by PCR and inserted in the pCRII vector by T/A cloning (Invitrogen). Cloned cDNAs were then inserted as EcoRI/XbaI or XhoI/XbaI fragments in pCMV-3×FLAG (Sigma, St Louis, MN) for expression in mammalian cells as a fusion with three FLAG epitope tags. The dominant-negative mutant of CDK9 (cdk9 D167N, pCMV cdk9-HA dn, [16] was a kind gift from A. Giordano (U.Penn., Philadelphia, USA). The *BRD4* cDNA fragment coding from residue 1 to 722 was cloned into pCMV-3xFLAG (Sigma). shRNA targeting *Af9* or *Brd4* mRNAs were cloned in pSIREN (Clontech) using the following oligonucleotides: *Brd4*: gatccgcctggagatgacatcgtcttattcaagagataagacgatgtcatctccaggttttttctcgagg, *Af9*: gatccgtgagtgtgcaaagacccaccttttcaagagaaaggtgggtctttgcacactcttttttttagatctg and their complementary counterparts to generate ecotrophic retroviruses according to the manufacturer instructions. Complete sequences are available on request. All constructs were verified by automatic sequencing (MWG Gmbh, Ebersberg, Germany). Other expression vectors were purchased from GeneCopoeia (Rockville, Md, USA).

### P19 Stable Clones Generation

Subconfluent P19 cells were transduced at MOI = 5 with lentiviral particles and further selected with puromycin. Individual clones were selected by the limited dilution method and characterized for the expression of either *Af9* or *Brd4*. Cell lines displaying a mRNA decreased expression above 80% were further characterized by western blotting. Initial experiments were carried out on two individual, independent subclones.

### Transient Transfections

HeLa or P19 cells were cultured as monolayers in Dulbecco’s modified Eagle’s medium supplemented with 10% fetal calf serum (Sigma). Transfections were carried out by the polyethyleneimine coprecipitation method with Exgen500 (Euromedex, Souffelweyersheim, FR) as described [11]. Twenty four hours after transfection, cells were treated overnight with 1 µM atRA and luciferase activity was quantified. Basal expression levels were arbitrarily set to 1 and data are expressed as the mean±SEM (n = 3–6). Luciferase assays were performed with the BrightGlo system (Promega, Madison, WI) and luciferase activity (as relative luciferase units, RLU) was measured with a Victor Light 1420 Luminescence counter (Perkin-Elmer, Waltham, MA).

### GST Pulldown Assays

**Figure 1:**
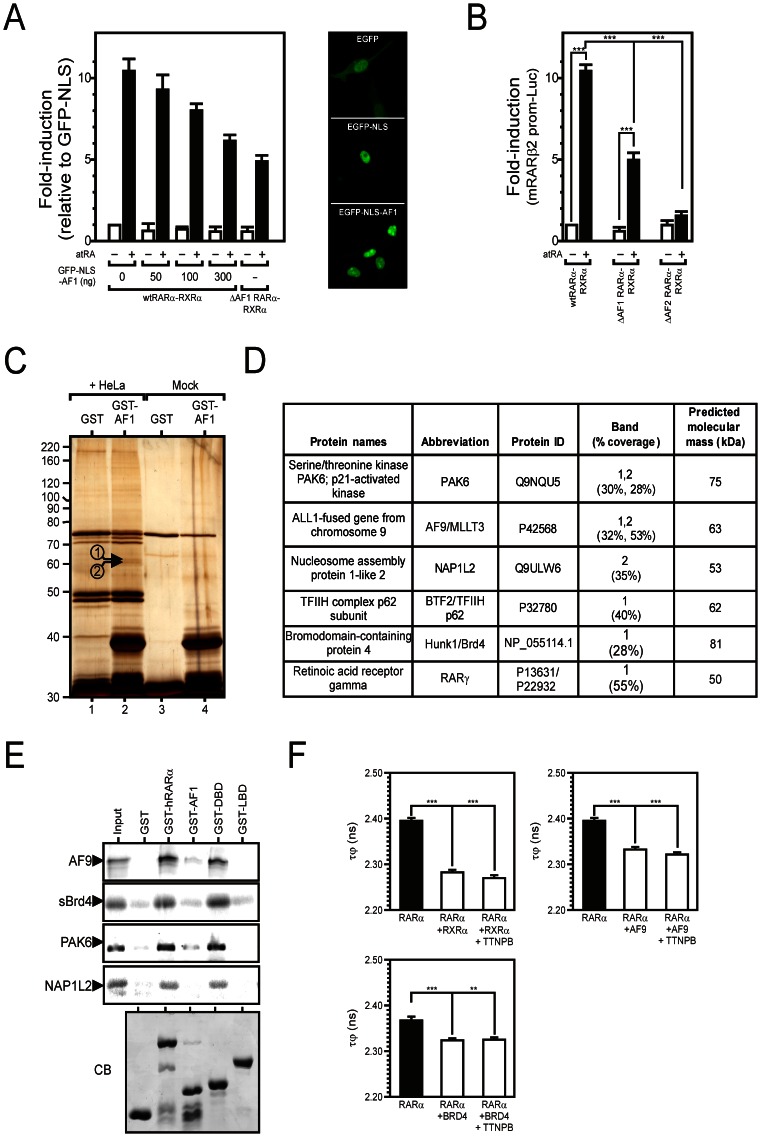
The N-terminus of RARα interacts with nuclear proteins. (A) A nucleus-targeted RARα AF-1 domain acts as a dominant negative receptor. HeLa cells were cotransfected with expression vectors coding for wild type (wt) RXRα, wtRARα, GFP-NLS and GFP-NLS-AF-1 at the indicated ratio together with a m*Rarβ2* promoter-driven reporter gene (mRARβ2-Luc). Cells were treated overnight with 1 µM atRA and luciferase activity was quantified. Basal expression levels were arbitrarily set to 1 and data are expressed as the mean±SEM (n = 3). *, p<0.05; **, p<0.01; ***, p<0.005. (Right panel) Confocal laser microscopy of transfected HeLa cells. (B) The RARα AF-1 domain is transcriptionally active. HeLa cells were transfected with mRARβ2-Luc and expression vectors coding for wtRXRα, wtRARα, N–terminally ΔAF-1-RARα) or C-terminally truncated ΔAF-2-RARα) RARα. Cell treatment, luciferase assays and calculations are as in (A). (C, D) Isolation and identification of proteins interacting with the AF-1 transactivation motif of RARα. AF-1 fused to GST (GST-AF-1) or GST alone (GST) were immobilized on a matrix and incubated with HeLa nuclear extracts (+HeLa) or buffer alone (Mock). Numbers indicate bands that were subjected to mass spectrometry analysis. (D) The table indicates the name, protein abbreviation, the UniProtKB/TrEMBL entry, percentage of peptide coverage in two representative purifications, and the predicted molecular mass. (E) Target validation by GST pulldown assays. Various domains of RARα were expressed as fusion proteins to GST (left panel) and used as baits for ^35^S-labeled shortBRD4 (sBRD4), AF9, PAK6 and NAP1L2. CB: Coomassie Blue staining of RAR derivatives adsorbed on glutathione-Sepharose. (F) Interaction of RARα with BRD4 or AF9. FLIM-based FRET fluorescence assays were performed to determine the lifetime of the donor (GFP) in the indicated conditions.

The following GST-RARα fusion proteins were used in these assays (Fig. 1E and Fig. S1A), with the numbers indicating the first and last amino acid of the RARα sequence: GST-hRARα (1–462); GST-AF-1 (1–92), GST-AF-1-DBD (1–158), GST-DBD (92–173); GST-DBD-LBD (92–462), GST-LBD (186–462). Glutathione S-transferase (GST) fusion protein expression and purification, and GST pulldown assays were performed according to Rachez et al. [17] with the following modifications. Immobilized GST fusion proteins were incubated in GST-binding buffer consisting of 20 mM Tris-HCl, pH 7.9, 0.2 mM EDTA, 0.1% Nonidet P (NP)-40, 0.5 mM PMSF, 1 mM DTT, protease inhibitors (Sigma), containing 1 mg/mL BSA and 100 mM KCl. Immobilized proteins on beads (20 µg) were incubated at 4°C for 6–10 hours with 2–6 mg of HeLa nuclear extract; or 2 µg proteins on beads were incubated at 4°C for 4 hours in the presence or absence of atRA (Sigma) with proteins synthesized by *in vitro* translation (TNT-coupled reticulocyte lysate, Promega) with ^35^S-methionine (GE-Healthcare, Waukesha, WI). After three washes in GST-binding buffer supplemented with 150 mM KCl and 0.3% NP-40, samples were resolved by SDS-PAGE, and detected either by silver nitrate staining or autoradiography followed by quantification with a Storm 860 phosphorimager (GE-Molecular Dynamics).

### Immunoprecipitation and Immunoblotting

Whole cell extracts from 10^5^ transfected HeLa cells were prepared in 0.25 mL lysis buffer [10 mM Tris·HCl, pH 7.5, 150 mM NaCl, 3 mM MgCl_2_, 0.5% NP-40, protease inhibitors (Sigma) and 0.1 U DNaseI (Sigma)]. After centrifugation, cleared extracts were diluted 5 times in lysis buffer without NP-40 and incubated at 4°C for 3 hours with 80 µL anti-FLAG M2 affinity resin (Sigma), or with the anti-RARα monoclonal antibody (Santa Cruz Biotechnology, Inc., Santa Cruz, CA) immobilized onto protein A sepharose (GE-Amersham) pre-equilibrated in lysis buffer. After four washes with lysis buffer adjusted to 0.15% NP-40, samples were resolved by SDS-PAGE and detected by western blot with appropriate primary antibodies, followed by incubation with HRP-coupled secondary antibodies and detection with ECL+ (GE-Amersham).

### Mass Spectrometric Analyses

Protein samples resolved by SDS-PAGE were detected by colloidal blue staining [17]. Bands were digested with trypsin and analyzed by Matrix-assisted laser desorption/ionization time-of-flight (Maldi-TOF) mass spectrometry. The SwissProt database was interrogated through the MS-Digest Search program [18].

#### Spot excision and in gel digestion

Protein spots were excised semi-automatically from 1D gels using the “Click and pick” mode of the Ettan Spot Picker (GE-Amersham Biosciences). Approximately ten plugs were excised from each band and put in the same well. In-gel digestion was performed using an automated protein digestion system (Ettan Digester; GE-Amersham Biosciences). Briefly, plugs were washed three times with 50 mM ammonium bicarbonate containing 50% methanol, then 50% acetonitrile containing 0.1% TFA. After two washing steps in 70% acetonitrile, plugs were dried and rehydrated with 10 µL of 20 mM ammonium bicarbonate containing 4 µg/mL trypsin (Promega) for 4 hours. Finally, a 20 mM ammonium bicarbonate solution was added overnight. The peptide mixture were then dried, and resuspended in 10 µL 0.1% TFA before MALDI-MS analysis.

#### Acquisition of mass spectrometric peptide maps, MALDI-TOF MS and database search and analysis

1 µL of peptide mixture was mixed with 1 µL of DHB matrix solution (10 mg dihydrobenzoic acid in 50% methanol) on the MALDI target. MALDI-TOF MS was performed using a Voyager DE STR mass spectrometer (PerSeptive Biosystems) equipped with a 337.1 nm nitrogen laser and the delayed extraction facility. All spectra were acquired in a positive ion reflector mode. Typically, 200 laser shots were recorded per sample, and spectra were internally calibrated (using the DataExplorerTM software, PerSeptive Biosystems) using three peptides arising from trypsin autoproteolysis ([M+H]+842,5100; [M+H]+1045.5642; [M+H]+2211.1046). Tryptic monoisotopic peptide masses were searched for in the NCBI, using Protein Prospector (http://prospector.ucsf.edu/) with a mass tolerance setting of 50 ppm, with three missed cleavage sites as fixed parameters, and with carbamidomethylation and methionine oxidation as variable modifications. The database search identified multiple proteins in each band. However, proteins to be further tested were chosen among the possible candidates by comparing the relative abundance of the different peptides, the percentage of recovery of each protein and taking into account the full size of the proteins. MS analysis was carried out 4 times on independent samples and selected peptides were detected at least twice out of 4 analysis, but in most cases they were detected 3 or 4 times out of 4 analysis. Proteins of interest were selected from this type of analysis if peptide coverage was above 20%. Specific samples were re-analyzed and provided enhanced spectra of protein digests which resulted in significantly increased sequence coverage (above 35%) and confidence in protein identification.

### RT-QPCR

RNAs were extracted as described in [11]. RT-QPCR analysis was carried out as described in [19]. When indicated, Taqman assayson demand (Life Technologies, Foster City, CA) where used: RARβ2 Mm01319674_m1 and Mm01319678_m15′; cdx1, Mm00438172_m1*and m00438173_gH; Hoxa1 Mm04208064_g1* and Mm00439359_m1; Stra8 Mm01165138_m1 and Mm00486473_m1*; HoxB4 Mm00657964_m1 and Mm01307004_mH.

### Microarray Analysis of mRNAs

Microrray hybridization and scanning were carried out following the manufacturer instruction (Agilent, One-color Microrray Gene Expression Analysis) using mouse SurePrint 8*60K arrays. Data processing and analysis were performed as described in [20] using the Genespring 12.0 software (Agilent). Briefly, array integrity was visually inspected, and quality controls were performed based on PCA analysis. Data were filtered to exclude signals in the low 20% and averaged. A gene-level analysis was performed and data filtered and graphed as detailed in the legend to figures. Data were deposited on the ArrayExpress web site with the accession number E-MEXP-3669.

### Chromatin Immunoprecipitation Assays

ChIP assays were carried out in duplicate as described in [11] using antibodies from Abcam (Cambridge, UK; AFF4, ab57077), Santa Cruz [TFIIH, sc293; cdk9, sc8339; cdk8, sc1521; RARα, sc551; RXRs, sc774; RNApol II, sc899; AF9(D17), sc32369; AF9(L15); sc32371; Brd4(H250), sc48772], Bethyl Laboratories (Montgomery, TX; AF9, A300-595A, A300-596A, A300-597A), Active Motif (Carlsbad, CA; Brd4, #39909). Bethyl antibodies were used as a 1∶1∶1 (vol:vol) mix in ChIP reactions; Brd4 was immunoprecipitated with a v:v mix of Santa Cruz and Active Motif antibodies. Results were acquired and quantified as described in [21] and [22].

### FRET Assays

Human RARα was cloned into pEGFP-C1 (Clontech, Palo Alto, CA, USA) to be expressed as a C-terminal EGFP-fusion protein. hRXRα, AF9 and Brd4 were cloned into the pReceiver-M55 backbone (Genecopoeia, Rockville, Md, USA) to generate mCherry-fusion proteins. Initial experiments showed that N-terminally fused mCherry proteins, but not C-terminally fused proteins, were suitable for FRET experiments together with the EGFP-RAR. HeLa cells were plated on 32 mm diameter glass coverslips 12 h before transfection with the FuGENE HD reagent (Roche Diagnostics, Rotkreuz, SW) according to the manufacturer’s recommendations. For FLIM-FRET imaging, the glass coverslips were deposited into a POC (Perfusion, Open and Close) chamber and the culture medium was replaced by Leibovitz’s 15 medium (L-15, Invitrogen). The frequency-domain FLIM measurements have been previously described in Leray et al [23]. The FLIM microscope is composed of a LIFA system (Lambert Instruments, Roden, The Netherlands) implemented on a spinning-disk confocal system (Yokogawa CSU-X1, Tokyo, Japan) adapted on a Leica (Lognes, FR) DMI6000B inverted microscope and equipped with a diode laser source emitting at a wavelength of 488 nm and whose intensity is modulated at 40 Mhz. Cells were imaged using a 63× oil-immersion objective (Leica HCX Plan Apo NA 1.4). Fluorescence emitted was then successively routed by a dichroic mirror (Semrock Di01-T405/488/568/647), spectrally filtered (Chroma, HQ545/30×) and detected with an intensified CCD camera (Li^2^CAM, Lambert Instruments) modulated at the same frequency (40 MHz) and coupled to an optical zoom (×2). The phase fluorescence lifetime for each sample was calculated from the acquisition of 36 phase-shift images using the LI-FLIM software (Lambert Instruments), after calibrating the system with a reference fluorescein solution at 1 µM of known lifetime (4 ns). For each set of acquisition, means and standard error of the means (S.E.M.) were determined from at least 8 samples per condition using the GraphPad Prism v5.0 software (San Diego, CA).

### Statistical Analysis

Values are reported as the mean±SEM, with 3–6 biological replicates (gene expression data). The statistical significance of differences amongst groups were determined using either a Student t-test (2 group) or ANOVA followed by a post-hoc test (Tukey) using GraphPad Prism v5.0. *, p<0.05; **, p<0.01; ***, p<0.005.

## Results

### RARα Interacts with Components of the SEC Complex

The AF-1 domain of RARα functions as an autonomous transactivation domain, as shown by squelching experiments in Hela cells using overexpressed, nuclear-targeted GFP fused to RARα AF-1 (Fig. 1A) and the defective transcriptional activity of RARα deleted from its N-terminal AF-1 domain (ΔAF-1-RARα, Fig. 1B), as previously reported in COS cells [24]. We thus identified novel RARα transcriptional coregulators using mass (MALDI-TOF) fingerprinting of HeLa nuclear proteins binding to the isolated RARα AF-1 domain (Fig. 1C and 1D). The p62 subunit of TFIIH (BTF2), known to interact physically and functionally with RAR AF-1 [25], [26] was isolated, thus validating our experimental strategy. Isolated proteins interacted with RARα in a ligand-independent manner, and included the novel potential coregulators PAK6 (p21-activated kinase-6), AF9/MLLT3 (ALL1 fused gene from chromosome 9), NAP1L2 (nucleosome associated protein 1-like 2) and the short isoform of BRD4 (70–80 kDa, sBRD4). Although full-length Brd4 has been described in HeLa cells as occurring as a 180–200 kDa polypeptide [27], all of the identified peptide sequences mapped within the sequence of sBRD4, in agreement with a number of reports describing BRD4 as a lower molecular mass polypeptide of ca. 70–80 kDa [28], [29].

Unexpectedly, we also identified RARγ as an AF-1 interactant. This suggested that RAR can homodimerize through this region and the LBD, a hypothesis which was confirmed by GST pulldown assays using labeled RARα deletion mutants and GST-hRARα AF-1. This assay indeed revealed that the RAR AF-1 interacts mostly with the LBD, hinting at inter-domain interactions (Fig. S1A). Therefore, our GST affinity matrix was constituted not only of the isolated AF-1, but also included full-length RARγ, and thus might have adsorbed proteins interacting, in a ligand-independent manner, not only with the isolated AF-1, but also with full length RARγ. Detected interactions were therefore validated using either the full length RARα or various RARα deletion mutants fused to GST as baits (Fig. 1E). All proteins interacted with full length RARα and showed a strong propensity to associate to the isolated RAR DBD. Preliminary experiments ruled out a possible contribution of the hinge region to the observed interactions (CR and PL, unpublished observations). PAK6, already described as an androgen and estrogen receptor corepressor [30], [31], and TFIIH were not considered further. Thus only AF9 interacted detectably with the isolated AF-1 domain, as shown by GST pulldowns and coimmunoprecipitation assays (Fig. 1E and Fig. S1B). AF9 and BRD4 were thus chosen for further study, as both potentiated RARα transcriptional activity without interacting with the RARα LBD (Fig. 1E), in contrast to NAP1L2, which, in addition, was inactive in the transcription assay (Fig. S1C). Molecular interactions were further assessed by fluorescence resonance energy transfer (FRET) in living Hela cells. The use of N-terminally tagged proteins (eGFP for RARα, mCherry for RXRα, AF9 and Brd4) showed a significant decrease of the donor lifetime (eGFP) coupled to RARα in the presence of the known RAR dimerization partner acceptor RXRα. Similarly, both AF9 and Brd4 induced a decrease of the donor lifetime, showing clearly that these proteins interact closely with RARα. Interactions were not modified upon treatment with a synthetic pan-RAR agonist (TTNPB), confirming that these interactions are ligand-independent (Fig. 1F).

### RARα-mediated Transcription is P-TEFB-dependent

**Figure 2:**
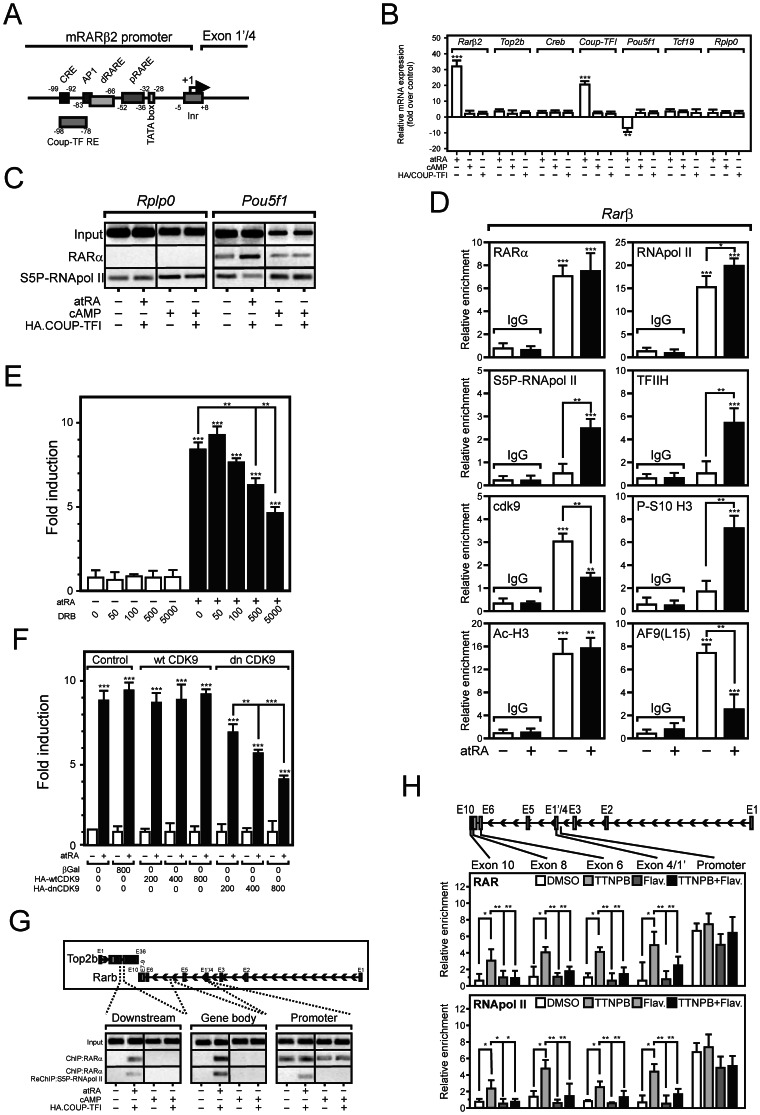
RAR localizes to transcribed regions of the Rarβ2 gene in a P-TEFB-dependent manner. (A) Structure of the mouse *Rarβ2* promoter. pRARE: proximal RARE; dRARE: distal RARE. (B) Gene expression in P19 cells. P19 cells were treated for 48 hours with DMSO, 1 µM all trans RA (atRA), 250 µM cAMP or transfected with a HA-tag COUP-TFI expression vector. Expression of *Rarβ2, Top2β*, *Creb*, *Coup-TFI*, *Tcf19* and *Rplp0* were quantified by RT-QPCR. Basal expression levels were arbitrarily set to 1 and data are expressed as the mean±SEM (n = 3). *, p<0.05; **, p<0.01; ***, p<0.005. (C) RARα and phosphorylated RNApol II loading at the *Rplp0* and *Pou5f1/Oct4* promoters. P19 cells were treated as in (B) and ChIP assays were performed with indicated antibodies. (D) AF9 colocalizes to the *Rarβ2* promoter. P19 cells were treated for 4 hours with 1 µM atRA, and ChIP assays were carried out as described. The specific enrichment in *Rarβ2* promoter sequence is expressed after normalization to background values (*Myoglobin* gene). Data are expressed as the mean±SEM (n = 2). *, p<0.05; **, p<0.01; ***, p<0.005. (E) DRB inhibition of the *Rarβ2* gene transcription. P19 cells were treated with the indicated combination of atRA (1 µM) and varying doses of DRB (50 to 5000 nM) for 4 hours. *Rarβ2* mRNA was quantified by RT-QPCR. (F) A CDK9 dominant negative mutant inhibits *Rarβ2* gene expression. P19 were cotransfected with increasing amount of pCMV-lacZ (control), pCMV-HA-wtCDK9, or pCMV-HA-dnCDK9 expression vectors at the indicated ratio, then treated 24 hours with 1 µM atRA. Gene expression was quantified as above and data expressed as the mean±SEM (n = 4). *, p<0.05; **, p<0.01; ***, p<0.005. (G) RAR and phosphorylated RNApol II are detected at transcribed regions of the *Rarβ2* gene. P19 cells were treated as in (D) and ChIP/reChIP assays were performed. (H) P-TEFb inhibition prevents RAR association to *Rarβ2* elongated regions. P19 cells were treated for 4 hours with the indicated combination of TTNPB (1 µM) or flavopiridol (250 nM). ChIP assays (n = 2) were performed as in (D).

P19 EC cells (noted P19^wt^ thereafter) are stem cell-like, pluripotent cells differentiating into neurons and glial cells upon atRA treatment [32]. In this cell line, as well as in other mouse EC cell lines, the *Rarβ2* promoter is the archetypical atRA-regulated promoter, harboring a consensus DR5 retinoic acid response element (RARE) and potential cis-acting motifs for COUP-TFI/NR2F1 and CREB (Fig. 2A). atRA, but not overexpressed COUP-TFI or cAMP, induced *Rarβ2* expression in P19 cells (Fig. 2B), showing that the DR5 RARE is a major functional cis-acting element. As expected, the mRNA expression of the pluripotency marker *Pou5f1/Oct4* was downregulated in response to atRA, whereas that of control genes *Top2β*, *Tcf19* and *Rplp0* was unaffected (Fig. 2B). In these conditions, a ligand-dependent binding of RARα to the negative RARE from the *Pou5f1* promoter [33] was detected by chromatin immunoprecipitation (ChIP) assays, performed with a specific anti-RARα antibody [11], [34] (Fig. 2C). RARα binding was concomitant to decreased RNA polymerase II (RNApol II) C-terminal domain (CTD) phosphorylation (S5P-RNApol II), a post-translational modification associated with promoter clearance and increased transcription. In similar conditions, S5P-RNApol II, but not RARα, associated to the retinoid-insensitive *Rplp0* ribosomal gene promoter (Fig. 2C). Thus the atRA-induced activity of the *Rarβ2* promoter depends mostly, if not exclusively, on RAR-driven events.

ChIP assays showed that RARα was constantly bound to the *Rarβ2* promoter, which harbored high levels of acetylated H3 histone irrespective of the presence of ligand (Fig. 2D and [6]), in agreement with the facilitating effect of histone acetylation on the binding of RAR-RXR heterodimers to nucleosomal DNA [10]. In contrast, a decreased density in the P-TEFb kinase subunit CDK9 and AF9 (Fig. 2D), known to associate to P-TEFb [35], was observed. Increased densities of phosphorylated histone H3 serine10 (P-S10 H3), of total (RNApol II) and of S5P-RNApol II were concomitant to *Rarβ2* activation, in agreement with the increased loading of the RNApol II kinase TFIIH (Fig. 2D and [11]).

P-TEFb catalyzes the phosphorylation of RNApol II on Ser2 and of the negative elongation factors NELF and DSIF [DRB (5,6-dichloro-1-β-D-ribofuranosylbenzimidazole) sensitivity-inducing factor] to promote the release of paused RNApol II [36]. DRB inhibited atRA-induced accumulation of *Rarβ2* mRNA in P19^wt^ with an EC_50_≈5 µM, suggesting that CDK9 activity is necessary for *Rarβ2* promoter activation (Fig. 2E). Similarly, increasing amount of a dominant-negative (dn) mutant of CDK9 (CDK9 D167N) blunted *Rarβ2* promoter activation (Fig. 2F). Thus the sensitivity of the *Rarβ2* promoter to CDK9 inhibition reflects the association of this chromatinized cis-acting sequence to the P-TEFb kinase CDK9 (Fig. 2D).

### Involvement of P-TEFB in Retinoid-mediated Transcription

The interaction of RARα with P-TEFb interactants raised the possibility that it could associate with elongating complexes. RARα association with various intronic or exonic regions of the *Rarβ2* locus was monitored by ChIP assays in P19 cells (Fig. 2G and 2H). RARα association with an intronic region occurred only after atRA treatment, and could also be observed, albeit to a much lower extent, downstream of the transcription termination site which mapped to the retinoid-insensitive 5′-flanking *Top2β* gene (Fig. 2G). ChIP-ReChIP assays demonstrated a colocalized, ligand-dependent accumulation of RAR and S5P-RNApol II on these transcribed regions (Fig. 2G). Thus RAR colocalizes with elongating RNApol II. To evaluate whether P-TEFb activity affects RAR association to transcribed regions of the *Rarβ2* gene, P19^wt^ were treated with TTNPB with or without the CDK9 inhibitor flavopiridol (Fig. 2H). While flavopiridol did not perturb RAR recruitment to the *Rarβ2* promoter and tended to decrease RNApol II density at this location, it clearly decreased the atRA-induced accumulation of RNApol II and of RAR at several exonic regions. Thus P-TEFb activity is required for RAR association to *Rarβ2* transcribed regions. This observation was unexpected in the light of a previous report showing that RAR is absent from exon 6 of the *Rarβ2* gene in atRA-treated HeLa cells [37]. Although BRD4 or AF9 are readily expressed in these fibroblastic cells, the high level of sequestration of P-TEFb in an inactive from in this rapidly proliferative cell line may explain this discrepancy [38]. Moreover, RAR displays a distinct binding kinetics with the RARβ2 RARE, which, in non-differentiating HeLa cells, cycles over time with a period of 4 hours [37] but decreases over time in EC F9 cells [39], thus pointing to distinct cell-specific transcriptional activation mechanisms.

### Functional Relevance of AF9 and BRD4/RARα Interaction in Retinoid-regulated Transcription

**Figure 3:**
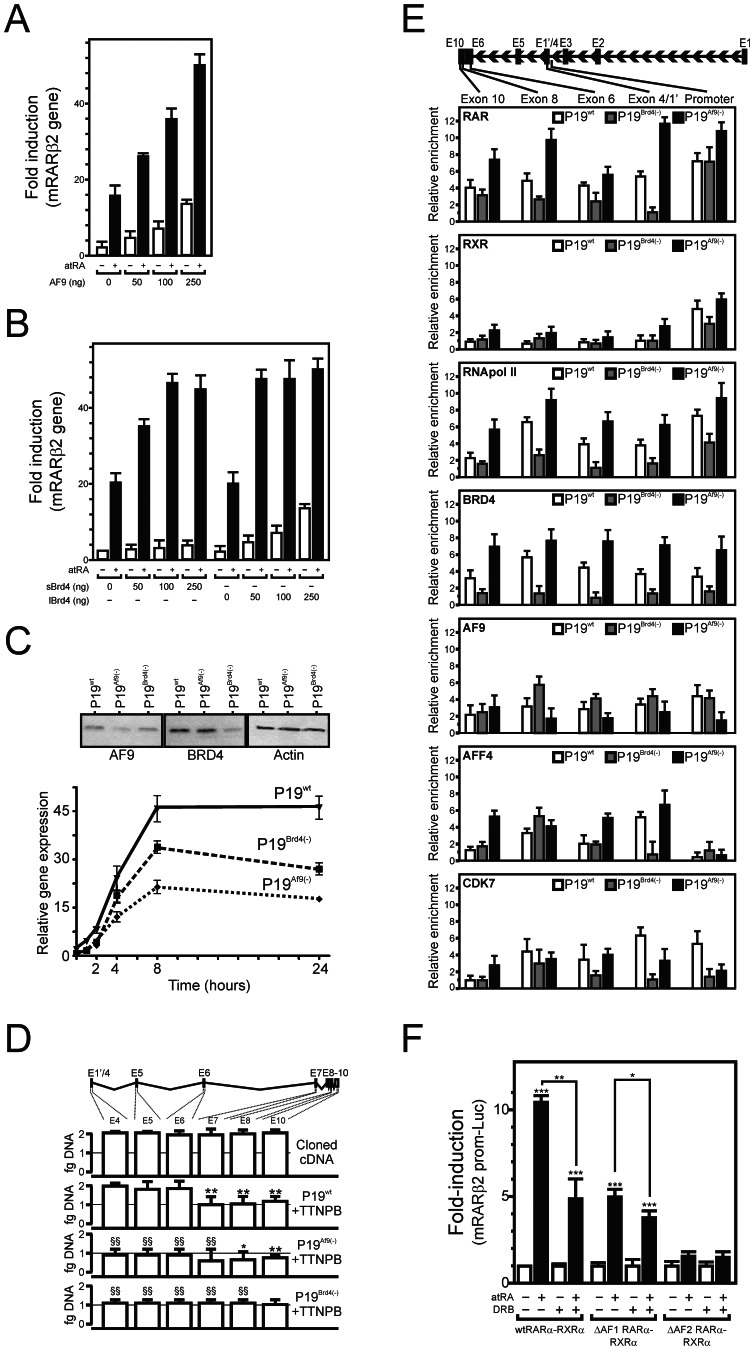
AF9 and BRD4 coactivate RARα in a ligand-independent manner. (A, B) P19 cells were transfected with the indicated amounts of AF9, sBRD4 or lBRD4 expression vectors for 24 hours with or without 1 µM atRA and *Rarβ2* gene expression was assayed by RT-QPCR. The basal expression level in non transfected, untreated cells was arbitrarily set to 1 and data were expressed as the mean±SEM (n = 5). *, p<0.05; **, p<0.01; ***, p<0.005. (C) *Af9* or *Brd4* knockdowns. (C, upper panel) AF9 or BRD4 expression was assayed by western blot analysis in P19wt, P19^Af9(−)^ and P19^Brd4(−)^. (C, lower panel) *Rarβ2* gene expression in AF9- or BRD4-depleted P19 cells. The time-dependent expression of *Rarβ2* upon stimulation with 1 µM TTNPB was quantified by RT-QPCR. (D) Exon-specific RT-QPCR assay of the *Rarβ2* mRNA. Cloned *mRarβ2* cDNA was used as a standard in PCR reaction, and used to select PCR primer sets displaying a similar efficiency (“Cloned cDNA”). *Rarβ2* mRNA from either P19^wt^, P19^Af9(−)^ or P19^Brd4(−)^ was then formally quantified by Q-PCR. **, p<0.01, intra-sample comparison; ^§§^, p<0.01, inter-sample comparison. (E) RAR associates to *Rarβ2* transcribed regions as a function of AF9 and BRD4 levels. P19^wt^, P19^Af9(−)^ or P19^Brd4(−)^ were treated with 1 µM TTNPB for 1 hour and ChIP assays were performed. The specific enrichment in the different *Rarβ2* amplicons was assayed by Q-PCR and expressed normalized to background values (myoglobin gene). Data are expressed as the mean±SEM (n = 2). *, p<0.05; **, p<0.01; ***, p<0.005. (F) The AF-1 region of RAR confers DRB sensitivity to RA-induced transcription of the *Rarβ2* promoter. P19 cells were cotransfected as indicated with expression vectors coding for wtRXRα, wtRARα or ΔAF-1-RARα or ΔAF-2-RARα together with the mRARβ2-Luc reporter gene. Cells were treated 24 hours with 1 µM atRA and/or DRB and luciferase activity was quantified. Basal expression levels were arbitrarily set to 1 and data are expressed as the mean±SEM (n = 6). *, p<0.05; **, p<0.01; ***, p<0.005.

AF9 overexpression strongly potentiated the basal expression level of the *Rarβ2* gene without affecting its ligand responsiveness in P19^wt^ (Fig. 3A), in agreement with its ligand-independent interaction with RARα (Fig. 1). The short isoform of BRD4 (sBrd4) exerted a significant effect on retinoid-induced RAR activity, whereas the full length BRD4 (lBrd4) was mostly active on the basal expression of the *Rarβ2* gene (Fig. 3B). Conversely, shRNA-mediated knockdown of *Af9* or of *Brd4* expression strongly blunted *Rarβ2* expression in P19 subclones [noted P19^Af9(−)^ and P19^Brd4(−)^, (Fig. 3C)]. To assess whether BRD4 and/or AF9 could play a role in transcription elongation, we assayed the abundance of *Rarβ2* mRNA using exon-specific PCR primer sets (Fig. 3D). After a 4 hour-induction, P19^wt^ accumulated about half the amount of full length transcript when compared to 5′ transcripts, suggesting a fast initiation process but a poor processivity. Amplification of exon sequences showed that the loss of processivity occurred between exon 6 and exon 7 of the *Rarβ* gene, which may relate to yet undefined particular chromatin structures. We noted that the loss of processivity was not significant in Brd4-depleted cells. In contrast, P19^Af9(−)^ and P19^Brd4(−)^ accumulated less 3′-truncated transcripts (or produced less abortive 5′ transcripts), suggesting that these AF9 and BRD4 act mainly on initiation or early elongation events.

### Transcription Elongation Factors and RAR Associate to the *Rarβ2* Gene Body

P-TEFb has been shown to bind to SEC, which contains the core component AFF4 and the coactivating AF9, or to BRD4 [38], [40]. TFIIH also participates in transcriptional elongation [41]. The recruitment of these factors to the *Rarβ2* locus, as well as that of RAR and RXR, was thus monitored after retinoid treatment (Fig. 3E). In P19^wt^, RNApol II was found within transcribed regions and at the promoter like the TFIIH subunit cdk7, whereas AFF4 density increased within the gene body. AF9 associated preferentially to the promoter region but was detected at significant levels at exonic regions, and BRD4 accumulated at the 3′ end of the gene. RAR was detected both at the promoter and exonic regions, whereas its heterodimeric partner RXR was surprisingly detected only at the promoter. P19^Brd4(−)^displayed a decreased RNApol II density throughout the *Rarβ2* locus, in agreement with the observed diminished *Rarβ2* mRNA synthesis. AFF4 and AF9 densities were barely affected, however cdk7 association was decreased. *Brd4* knockdown also impacted negatively on RAR loading in elongated regions. In contrast, *Af9* silencing globally increased RNApol II, BRD4, RAR and, to a lower extent, AFF4 loading. This suggested that AF9 exerts a negative effect on BRD4 recruitment, and that the observed decreased transcriptional activity of *Rarβ2* upon AF9 depletion may correlate with decreased RNApol II processivity. Importantly, the cdk9 inhibitor DRB significantly blunted the transcriptional activity of full length RARα in the presence of atRA, but was much less efficient on the transcriptional activity of the N-terminally truncated RARα (ΔAF-1-RARα, Fig. 3F), suggesting that AF-1 integrity is required to confer P-TEFb dependency to RAR-mediated transcription.

### AF9 and BRD4 Regulate Distinct Retinoid-regulated Gene Clusters

Retinoids trigger neuronal differentiation of P19 cells by regulating a network of RAR-driven genes. Indeed, 188 and 66 genes were up- or down-regulated respectively by more than 2-fold after a 4 hour-treatment of P19 embryoid bodies with atRA (Fig. S2A and Table S2). Functional annotation of these genes identified clusters of genes involved in embryo development and patterning (Fig. S2B), showing that P19 cells recapitulate initial transcriptional events leading to neuronal differentiation. For example, genes from the *Hox* cluster were significantly upregulated, as well as *Cyp26a1*, involved in the catabolism of RA.

We first asked whether BRD4 or AF9 played a role in controlling gene basal expression levels. While 31,948 genes were significantly expressed in P19^wt^, only 397 or 459 genes were up- or down-regulated by more than 5-fold in untreated P19^Brd4(−)^ or P19^Af9(−)^, respectively (Fig. S3). Among these 2 sets of genes, *Hoxa4* was the only known retinoid-target gene to be down-regulated in both cellular backgrounds (Table S2), showing that these two elongation factors do not contribute to basal transcription of retinoid-regulated genes.

**Figure 4:**
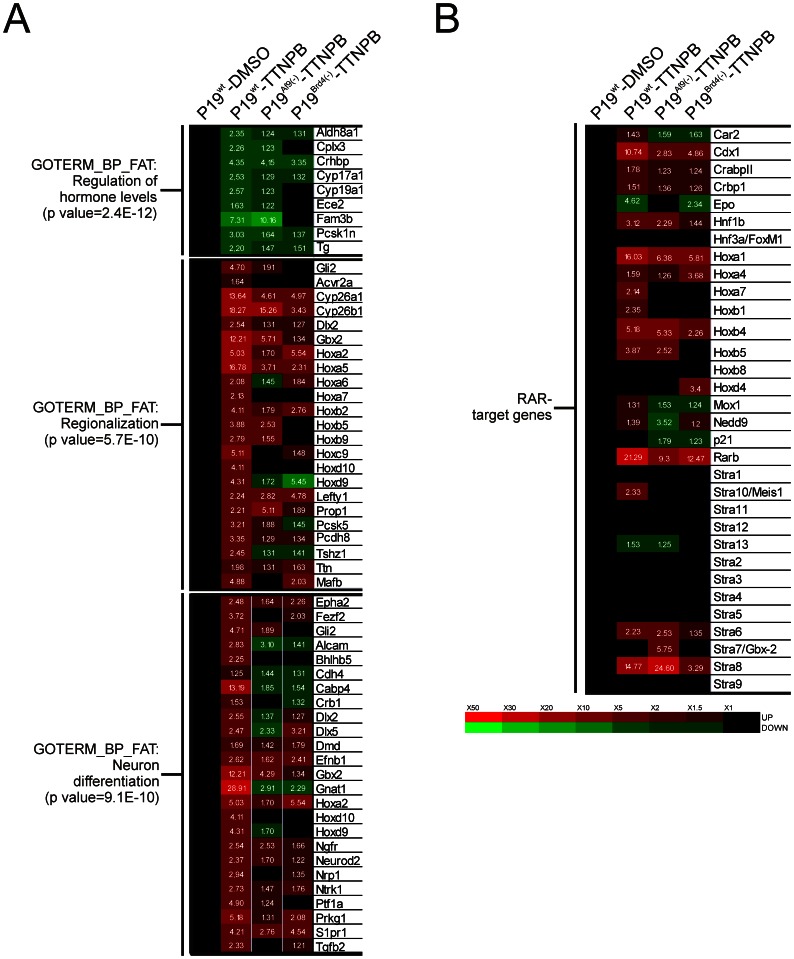
Gene expression level in response to RARα activation in wild type, AF9- and BRD4-deficient backgrounds. (A) Genes exhibiting a fold-change above 1.2 fold in TTNPB-treated P19^wt^ cells were clusterized according to a functional gene ontology classification. Representative functional clusters from the top 10 hits are shown. The basal level in non treated P19^wt^ was arbitrarily set to 1 and is depicted by black boxes. Numbers indicate the fold change ratio of individual genes relative to untreated wt P19 (red: upregulation; green: downregulation; black, no change). (B) Gene expression levels of known atRA-target genes. RA-target genes were selected from the literature and their expression levels were extracted from microarray data. Results are represented as in (A).

Gene expression levels were determined after a 4-hour treatment of P19^wt^ with the RAR-specific agonist TTNPB. The gene program induced by this synthetic ligand was very similar to that induced by atRA (91% overlap, Fig. S4 and Table S3). Gene expression levels in stimulated conditions (TTNPB, 4 hours) were calculated for each cellular background and expressed as fold-change over basal levels in P19^wt^ set to 1 for each gene (Fig. 4A and 4B). While examination of the gene expression pattern (Fig. S5A) showed a limited perturbation of the P19^wt^ transcriptome, a gene-by-gene analysis revealed that genes involved in cellular differentiation displayed a differential sensitivity to BRD4 or AF9 levels (Fig. 4A). Importantly, many known atRA-target genes were selectively affected by AF9 or BRD4 silencing (Fig. 4B), pointing at distinct functions of these two elongation factors in atRA-induced transcription. This conclusion was validated by the fact that AF9 depletion did not affect P19 cell differentiation, as assayed by the increased expression of the 160kDa neurofilament, while *Brd4* knockdown blocked this process (Fig. S5B).

**Figure 5:**
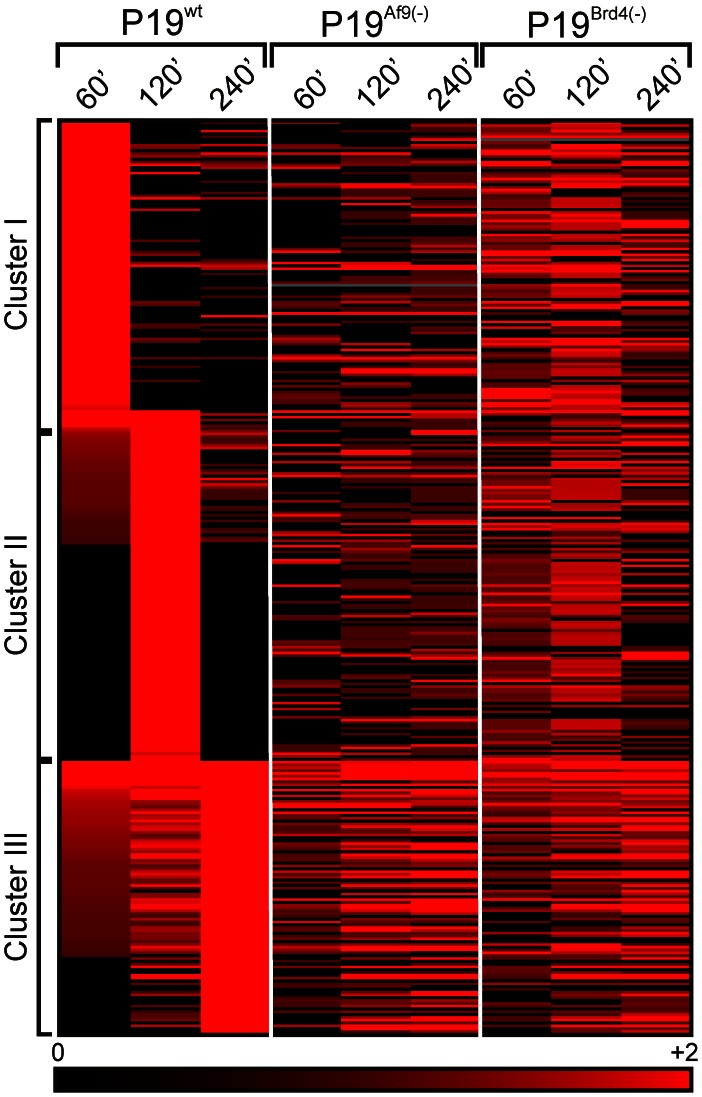
Time-dependent induction of gene expression upon RARα activation in P19^wt^, P19^Af9^^(−)^ or P19^Brd4^^(−)^. Cells were treated with TTNPB for indicated times and gene expression patterns were monitored. Genes induced more than 2-fold and peaking at either 60 minutes, 120 minutes or 240 minutes in the P19^wt^ background were clusterized to define cluster I (peaking at 60 minutes), cluster II (peaking at 120 minutes) and cluster III (peaking at 240 minutes). Associated gene lists were used to generate entity lists in Genespring to follow the expression of these genes in the P19^Af9(−)^ or P19^Brd4(−)^ background. Expression at different times in distinct cellular backgrounds is displayed as a heatmap.

BRD4 has been recently described as having a limited contribution to the rapid induction of a subset of retinoid-regulated genes, in contrast to SEC which had a broad impact on this process in mouse ES cells [35]. We thus investigated whether BRD4 or/and AF9 could contribute directly to the rapid induction of RAR-controlled genes in P19 cells. Genes displaying a maximal expression (FC>2) after a 60 min.(cluster I, Fig. 5), 120 min. (cluster II, Fig. 5) or 240 min. (cluster III, Fig. 5) treatment with TTNPB were identified in P19^wt^ cells. The expression of these clusters was monitored in P19^Brd4(−)^ or P19^Af9(−)^ to show that early-induced genes (clusters I and II) were more sensitive to *Brd4* or *Af9* knockdown than genes from cluster III. Thus both BRD4 and AF9 affect differentially atRA-regulated gene expression in our system, and favor the rapid induction of a limited set of genes.

### AF9- and BRD4-independent Genes Do Not Recruit RAR to Promoters and Transcribed Regions

**Figure 6:**
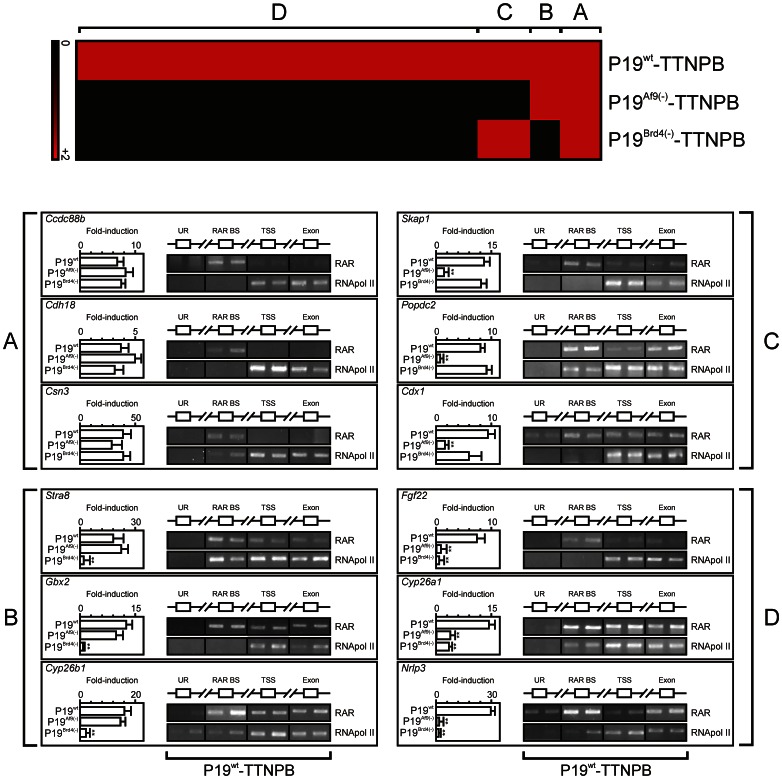
RARα association to transcribed regions in AF9-or BRD4-independent genes. The response of TTNPB-inducible genes (FC>2 after 4 hours) in P19^wt^ was compared to that in P19^Af9(−)^ or P19^Brd4(−)^ in similar conditions. Genes losing their responsiveness to TTNPB (FC<1.2) in either the P19^Brd4(−)^ background (cluster B), the P19^Af9(−)^background (cluster C) or both (cluster D) were identified by microarray data analysis. Genes maintaining an inducibility similar to that observed in P19^wt^ in either the P19^Af9(−)^ or the P19^Brd4(−)^ background were grouped in Cluster A. Genes in each cluster were searched for the occurrence of RAR binding sites on the basis of RAR ChIP-Seq data carried out in mouse ES cells [42]. Three representative genes were selected from each cluster and their inducibility was validated by RT-QPCR in each condition (n = 3, left inset). RARα and RNApol II association to an upstream region (UR), RAR binding site (RAR BS), transcriptional start site region (TSS) and an exon (Exon) was assessed in independent, duplicate ChIP-PCR assays after a 4-hour challenge of P19^wt^ with TTNPB. Input lanes showed an equal loading but were omitted for space purposes.

A close examination of the gene expression pattern by fold expression over time (Fig. 4D) also showed that some retinoid-regulated genes were insensitive to BRD4 or AF9 depletion. Gene expression data were therefore organized in 4 clusters of genes maintaining, or not, a significant induction rate (more than 50% of the gene activity in the wild type background) upon TTNPB treatment (4 hours, Fig. 6, top panel) in P19^Brd4(−)^ or P19^Af9(−)^ backgrounds. Four clusters were defined on the basis of gene induction sensitivity to BRD4 or AF9 depletion (AF9 and BRD4-independent, cluster A; BRD4-dependent and AF9-independent, cluster B; AF9-dependent and BRD4-independent, cluster C; AF9- and BRD4-dependent, cluster D). Three genes within each cluster harboring a RAR binding site within 30 kb of the gene boundaries as documented in mouse ES cells (Table S1, [42]) were selected for further characterization. The recruitment of RAR and of RNApol II was monitored by ChIP-PCR assays, due to the length of selected sequences, in TTNPB-treated P19^wt^ cells at either a control upstream region devoid of potential RARE (UR), at the identified RAR DNA binding site (RAR BS), at the transcription start site (TSS) or within an exon without potential RARE (Exon, Fig. 6). Control UR associated neither to RAR nor to RNApol II in these conditions, while RAR was consistently detected at RAR binding sites. RNApol II accumulated at TSS and exonic regions, as expected from increased mRNA transcripts synthesis (left inserts, Fig. 6). In contrast to AF9- and/or BRD4-dependent genes (clusters C and B respectively), RAR association to either TSS or exonic regions was not detectable for AF9- and BRD4-independent genes (cluster A, *Ccdc88b*, *Cdh18*, *Csn3*), suggesting that these factors are required for RAR recruitment, or at least stable binding, to elongated regions. It also suggested that the retinoid-mediated activation of BRD4 and/or AF9-dependent genes differs significantly from those exhibiting no such dependency, as emphasized by the selective accumulation of RAR at the TSS from BRD4- and AF9-dependent genes.

### AF9 or BRD4 Stabilize RAR Interaction with Transcribed Regions

**Figure 7:**
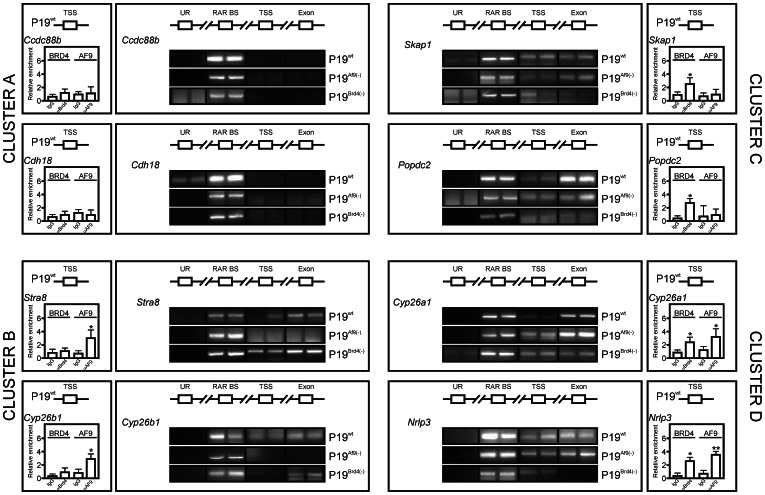
AF9 or BRD4 are required for RAR interaction with exonic regions. Two representative genes from clusters defined in Figure 5 were selected. The loading of AF9 and BRD4 to the TSS was assayed by ChIP-QPCR (n = 2) and results normalized to background values (*Myoglobin* gene) are represented in left insets (bar graphs). The association of RAR to an upstream region (UR), RAR binding site (RAR BS), transcriptional start site region (TSS) and an exon (Exon) was assessed in independent, duplicate ChIP-PCR assays after a 4-hour challenge of P19^wt^, P19^Brd4(−)^ or P19^Af9(−)^ cells with TTNPB. Data are expressed as the mean±SEM (n = 2). *, p<0.05; **, p<0.01.

Given that RAR did not associate to exonic regions of genes from cluster D (ie AF9- and BRD4-independent), we characterized RAR occupancy in the P19^Brd4(−)^ or P19^Af9(−)^ background (Fig. 7). In good agreement with gene expression data, ChIP-QPCR assays revealed that TSS occupancy by either BRD4 or AF9 could be detected at BRD4 and/or AF-dependent genes (clusters B, C and D), but not at the TSS of BRD4- and AF9-independent genes (cluster A). Silencing of *Af9* or of *Brd4* did not modify RAR loading at any of the locations tested in genes from cluster A. RAR loading in AF9-dependent genes (cluster B) was not affected by BRD4 depletion. In contrast, *Af9* knockdown impaired RAR association to exonic regions, whereas binding to the “RAR BS” was not significantly affected. BRD4-dependent genes (cluster C) mirrored this response, since only *Brd4* silencing affected RAR density at exonic sequences. Furthermore, AF9- and BRD4-dependent genes reflected the behavior of the *Rarβ2* gene, for which *Brd4* silencing reduced RAR association to transcribed regions and *Af9* knockdown had an opposite effect. As BRD4 and AF9 were shown to belong to distinct complexes and to interact with RAR DBD, their partitioning was assessed by coimmunoprecipitation of endogenous proteins from P19 cells. These assays showed that while immunoprecipitation of RARα isolated a fraction of BRD4 or of AF9, immunoprecipitation of AF9 was exclusive of that of BRD4, and vice-versa (Fig. S6). These experiments show that either BRD4 or AF9 is sufficient for RAR association to transcribed regions, and suggests that AF9 has an inhibitory role on RAR-BRD4 association.

## Discussion

In the current study, mass spectrometry-fingerprinting was used to identify AF-2-independent RARα coactivators. The unexpected interaction of RARα with RARγ led to the identification of BRD4 and AF9 as interacting strongly with the DNA-binding domain of RARα, and to a lesser extent with the N-terminal AF-1 domain in the case of AF9. Using the *Rarβ2* promoter as a paradigm for RAR-mediated transcription, we demonstrate that both proteins displayed RARα coactivating properties, thus functionally and physically connecting RAR to P-TEFb (cyclinT:CDK9), which interacts in an exclusive manner with BRD4 or AF9 [38]. In addition, we observed that RARα can associate to transcribed regions together with RNApol II. CDK9 catalyzes RNApol II CTD Ser2 phosphorylation, allowing the release of paused RNApol II complex for productive RNA elongation by relieving the inhibitory action of the DRB sensitivity-inducing factor DSIF and of the negative elongation factor NELF [reviewed in [36]]. Both the pharmacological inhibition of the P-TEFb kinase CDK9 and AF9 depletion blunted *Rarβ2* gene transcription. RNApol II processivity is controlled by AF9, which directly or indirectly regulates positively CDK9 activity [43]. Thus a decreased RNApol II processivity in *Af9*-depleted P19 cells (Fig. 3E) may account for the increased RAR and RNApol II association to gene body sequences and the observed decreased gene transcription.

BRD4 is known to bind to both components of P-TEFb, cyclinT and cdk9 [27]. It preferentially associates to P-TEFb at the promoter-proximal regions of Ca^2+^- or of HIV1-regulated genes and is excluded from AFF4-containing complexes [43]–[47]. BRD4 is however required for the induction of the HIV-1 promoter [8], and show a preferential activity on primary response genes [48]. The negative impact of *Brd4* silencing on some rapidly induced genes in our system (Fig. 5) is in agreement with this observation and suggests that retinoid-controlled transcription may depend either on the SEC or on the BRD4 version of P-TEFb. Interestingly, BRD4 is required for the rapid induction of a subset of retinoid-regulated genes (our study and [35]), and favors P-TEFb loading due to its affinity for phosphorylated H3Ser10 and acetylated histones H3 and H4 [7], [27], [49]. Importantly, all of these epigenomic marks are present at the archetypical *Rarβ2* promoter, whose expression is dependent on BRD4 levels (our study and [11]). FRET studies showed convincingly that RARα interacted directly not only with AF9 but also with BRD4 in living cells, suggesting that RARα may constitute a landing pad for BRD4 on retinoid-activated promoters.

An unexpected finding was that some retinoid-regulated genes, including *Rarβ2*, display a similar dependency on BRD4 and AF9 expression. The mutually exclusive binding of P-TEFb to BRD4 and AF9, as well as the similar binding interface on RARα, rules out the co-occurrence of BRD4 and AF9 into the same complex. The observation that AF9 depletion favors BRD4 and RAR recruitment to transcribed regions, yet promotes a decreased transcription efficacy, suggests that BRD4 plays, for this specific cluster (Fig. 6, Cluster D), a moderate role in transcriptional regulation. This is in line with the reported broad distribution, but restricted regulatory role of BRD4 in ES retinoid-regulated genes [35].

The selective recruitment of RAR, but not of RXR, to transcribed regions depending on P-TEFb activity is an unprecedented observation. The functional role of this association is likely to increase transcriptional efficiency, as shown by the loss of rapid gene induction upon *Brd4* or *Af9* knockdown and RAR association to gene bodies. Indeed, the *Hoxb1* gene, which is poorly inducible in our system, is weakly activated in mouse ES cells and does not recruit the SEC complex upon retinoic acid challenge [35]. In contrast, both *Hoxa1* and *Cyp26a1* are strongly induced in both systems and recruit SEC in mouse ES cells. This correlates SEC loading to rapid transcriptional induction, and both AF9 and BRD4 depletion preferentially affected early-induced genes in our model. Our present work, together with published data [50]–[52], thus show that nuclear receptors can engage physical and functional interaction with the transcriptional elongation machinery, thus providing a mechanistic basis for SEC targeting to conditionally-activated promoters.

In this study, we thus report the following original findings: (i) the P-TEFb interactants BRD4 and AF9 bind directly to and coactivate RAR in living cells; (ii) the activation of the archetypal retinoid-inducible gene *Rarβ2* is dependent on P-TEFb activity; (iii) RAR, but not RXR, associates to transcribed regions of the *Rarβ2* locus in a cdk9-dependent manner and colocalizes with elongating RNApol II; (iv) BRD4 and AF9 facilitate retinoid-induced transcription and exert distinct biological functions in retinoid-mediated neuronal differentiation. Thus monomeric RAR may be involved in promoter-pausing release and possibly transcriptional elongation as shown by our data. Such a dual function has been suggested recently for the transcription factor c-Myc [53]. Whether the RAR/elongation complex serves structural and/or functional functions call for further investigations, which will require to take into account the multiple functions of components of this multimeric complex and the use of simplified transcription systems.

## Supporting Information

Figure S1 RAR interaction with isolated RAR domains and functional interference with NAP1L2. (A) Various domains of RARα were expressed as fusion proteins to GST (left panel) and used as baits for ^35^S-labeled RARα AF-1. Protein bound on beads are visualized by autoradiography, in comparison to 10% input (Input). CB: Coomassie Blue staining of RAR derivatives adsorbed on glutathione-sepharose beads. (B) Coimmunoprecipitation of AF9 and RARα in HeLa cells. HeLa cells were cotransfected with expression vectors coding for either wtRARα (wt) or N-terminally truncated RAR (ΔAF-1) together with an empty pCMV-3×FLAG plasmid (Mock), or pCMV-3×FLAG containing a AF9 cDNA insert. Cell lysates were immunoprecipitated (IP) with an anti-FLAG M2 affinity resin, and immunoprecipitates, as well as cell lysates (Input), were analyzed by western blotting with an anti-RAR antibody. The numbers (ratio) are the ratio of RAR to AF9 detected by western blotting and quantified by densitometric analysis. (C) P19 cells were transfected with the indicated amounts of a NAP1L2 expression vector for 24 hours and *Rarβ2* gene expression level was assayed after a 4-hour treatment with 1 µM atRA, using a Taqman-based RT-QPCR assay. The basal expression level in untreated cells was arbitrarily set to 1 and data are expressed as the mean±SEM (n = 3). *, p<0.05; **, p<0.01; ***, p<0.005.
(TIFF)

Figure S2 Retinoic acid induces a neuronal differentiation program in EC P19 cells. (A) Microarray gene expression analysis of RA-stimulated P19 cells. A mRNA expression scatter plot was obtained from gene-level interpretation of microarray data (left panel), out of which the 10 most up- or down-regulated genes were identified (right panel). The two thick green lines in the scatter plot indicate a fold change greater than 2. (B) Genes whose expression was modulated more that 2-fold were clustered using the Gene Ontology functional annotation table [1].
(TIFF)

Figure S3 Basal gene expression in Af9- or Brd4-depleted P19 cells. P19^wt^, P19^Brd4(−)^ or P19^Af9(−)^ cells were treated for 4 hours with 100 nM TTNPB, and mRNAs were extracted and analyzed on Agilent microarrays. Basal expression level was set to 1 in the P19^wt^ background, and genes deregulated by more than 2-fold in both P19^Brd4(−)^ or P19^Af9(−)^ backgrounds were identified using the Genespring software. An entity list consisting of all genes displaying an altered expression in either P19^Brd4(−)^ or P19^Af9(−)^ cells was generated, and their expression level in each background was extracted from microarray data. These expression values, expressed as fold-change over basal in P19^wt^ cells, were processed and used to generate heatmaps with MeV [2]. Genes displaying a FC>5 in the P19^Brd4(−)^ background (top) or in the P19^Af9(−)^ background (bottom) were analyzed using Gene Ontology Functional Annotation Tables and the KEGG database [1]. The most significant terms are indicated. Finally, genes displaying a FC>5 in the P19^Brd4(−)^ background or in the P19^Af9(−)^ background were compared and genes similarly affected by both knockdowns were identified, and subjected to GO FAT and KEGG annotations. These analyses appear in the middle of the figure. Green: down-regulated; red: up-regulated. Raw data are available in Table S3.
(TIFF)

Figure S4 atRA and TTNBP elicits a similar transcriptional program in EC P19 cells. Genes that were modulated with a FC>1.5 at least in one condition (1 µM atRA or 1 µM TTNPB) were identified by data analysis in Genespring v12.0 and the gene list was exported as a text file from this software (available in Table S2). A partial heat map corresponding to upregulated genes (FC>2 in atRA-treated P19) classified by order of induction was generated from this worksheet using MeV and is shown fragmented for visualization purposes from left to right. Up- or downregulated genes (FC>2) are indicated in red or green respectively.
(TIFF)

Figure S5 Gene expression induction in an Af9- or Brd4-deficient cellular background. (A) Scatter plot representation of the gene expression pattern in wild type P19 (P19^ wt^), AF9-depleted P19 or BRD4-depleted P19 (P19^Af9(−)^ or P19^Brd4(−)^ respectively). The two thick green lines in scatter plots indicate a fold change greater than 2. (B) The expression of the neuronal differentiation marker NF160 was assessed by western blot analysis 96 hours after the initiation of neuronal differentiation as previously described [3].
(TIFF)

Figure S6 BRD4 and AF9 binds to RAR as distinct complexes. (A) Western blot of P19 whole cell extracts (100 µg proteins). P19 cells were grown under standard conditions and whole cell extracts were prepared as described in the Materials & Methods section. Proteins were resolved on 4–15% polyacrylamide gels and analyzed by western blotting using an anti-RAR (Santa Cruz, sc551), an anti-Brd4 (Active Motif) or an anti-AF9 (Bethyl A300-595A) antibody. (B) Coimmunoprecipitations assays. RAR was immunoprecipitated with an anti-RAR antibody (Santa-Cruz, sc551). Isolated complexes were resolved by 8% SDS-PAGE and analyzed by western-blotting using an anti-Brd4 (Active Motif) or an anti-AF9 antibody (Bethyl A300-595A). Reciprocal immunoprecipitations were carried out using an anti-Brd4 antibody (Santa Cruz H250) or a mix of anti-AF9 antibodies (Bethyl Labs). Input: 10% of total material (500 µg proteins); IgG: non-immune sera (rabbit); IP: immunoprecipitation with the indicated antibody.
(TIFF)

Table S1 Gene chromosomal coordinates and associated regions amplified by PCR in ChIP experiments. * Taken from [4]. **TSR coordinates are extracted from databases using Genomatix Eldorado. TSR amplicons were defined by extending the identified TSR with +/−50 bp. RARE motif search were carried out using this extended sequence and PCR primers were designed to optimally amplify this sequence using Primer3plus. When multiple potential RARE were detected, primers were designed to encompass all RAREs whenever possible. *** as defined by MathInspector scanning of the sequence using the V$RXRF matrix.
(XLSX)

Table S2 Gene basal expression in the P19^wt^, P19^Brd4(−)^ or P19^Af9(−)^ background. Genes that were modulated with FC>2 at least in one condition (shAF9 or shBrd4 vs wtP19) were identified by data analysis at the gene level in Genespring v12.0. The gene list was exported as a text file from this software. Gene expression levels in wtP19 were set to arbitrarily set to 1 and gene expression levels observed in either the AF9- or the BRD4-depleted background were expressed relative to this control. A heatmap corresponding to up- or down-regulated genes were generated from this worksheet using MeV and appears in the Supporting information files (Fig. S3).
(XLSX)

Table S3 atRA and TTNPB elicit a similar transcriptional response in P19 EC cells. Genes that were modulated with FC>1.5 at least in one condition (1 µM atRA or 1 µM TTNPB) were identified by data analysis in Genespring v12.0 and the gene list was exported as a text file from this software. Up- or downregulated genes (FC>2) are indicated in red or green respectively. A partial heat map corresponding to upregulated genes was generated from this worksheet using MeV and appears in Figure S3.
(XLSX)

Text S1 References used and cited in the Supporting Information Files.
(DOCX)
