# Supplementary figures and images for: The Elongation Complex Components BRD4 and MLLT3/AF9 Are Transcriptional Coactivators of Nuclear Retinoid Receptors
Source: PLoS One. 2013 Jun 10;8(6):e64880. doi: 10.1371/journal.pone.0064880 (PMC3677938; doi:10.1371/journal.pone.0064880)

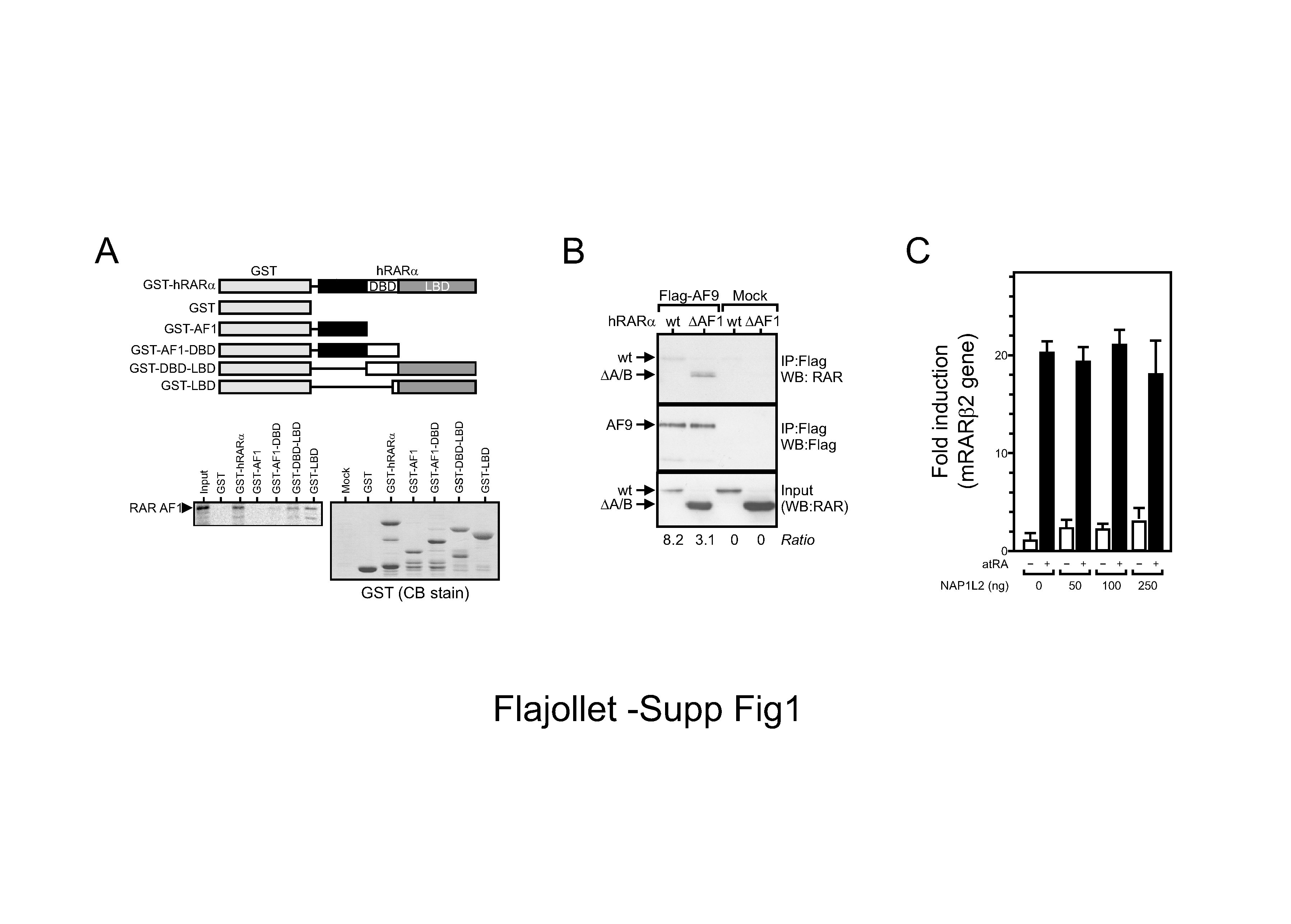

Supplement: Figure S1 — RAR interaction with isolated RAR domains and functional interference with NAP1L2. (A) Various domains of RARα were expressed as fusion proteins to GST (left panel) and used as baits for 35S-labeled RARα AF-1. Protein bound on beads are visualized by autoradiography, in comparison to 10% input (Input). CB: Coomassie Blue staining of RAR derivatives adsorbed on glutathione-sepharose beads. (B) Coimmunoprecipitation of AF9 and RARα in HeLa cells. HeLa cells were cotransfected with expression vectors coding for either wtRARα (wt) or N-terminally truncated RAR (ΔAF-1) together with an empty pCMV-3×FLAG plasmid (Mock), or pCMV-3×FLAG containing a AF9 cDNA insert. Cell lysates were immunoprecipitated (IP) with an anti-FLAG M2 affinity resin, and immunoprecipitates, as well as cell lysates (Input), were analyzed by western blotting with an anti-RAR antibody. The numbers (ratio) are the ratio of RAR to AF9 detected by western blotting and quantified by densitometric analysis. (C) P19 cells were transfected with the indicated amounts of a NAP1L2 expression vector for 24 hours and Rarβ2 gene expression level was assayed after a 4-hour treatment with 1 µM atRA, using a Taqman-based RT-QPCR assay. The basal expression level in untreated cells was arbitrarily set to 1 and data are expressed as the mean±SEM (n = 3). *, p<0.05; **, p<0.01; ***, p<0.005. (TIFF) [file pone.0064880.s001.tiff]

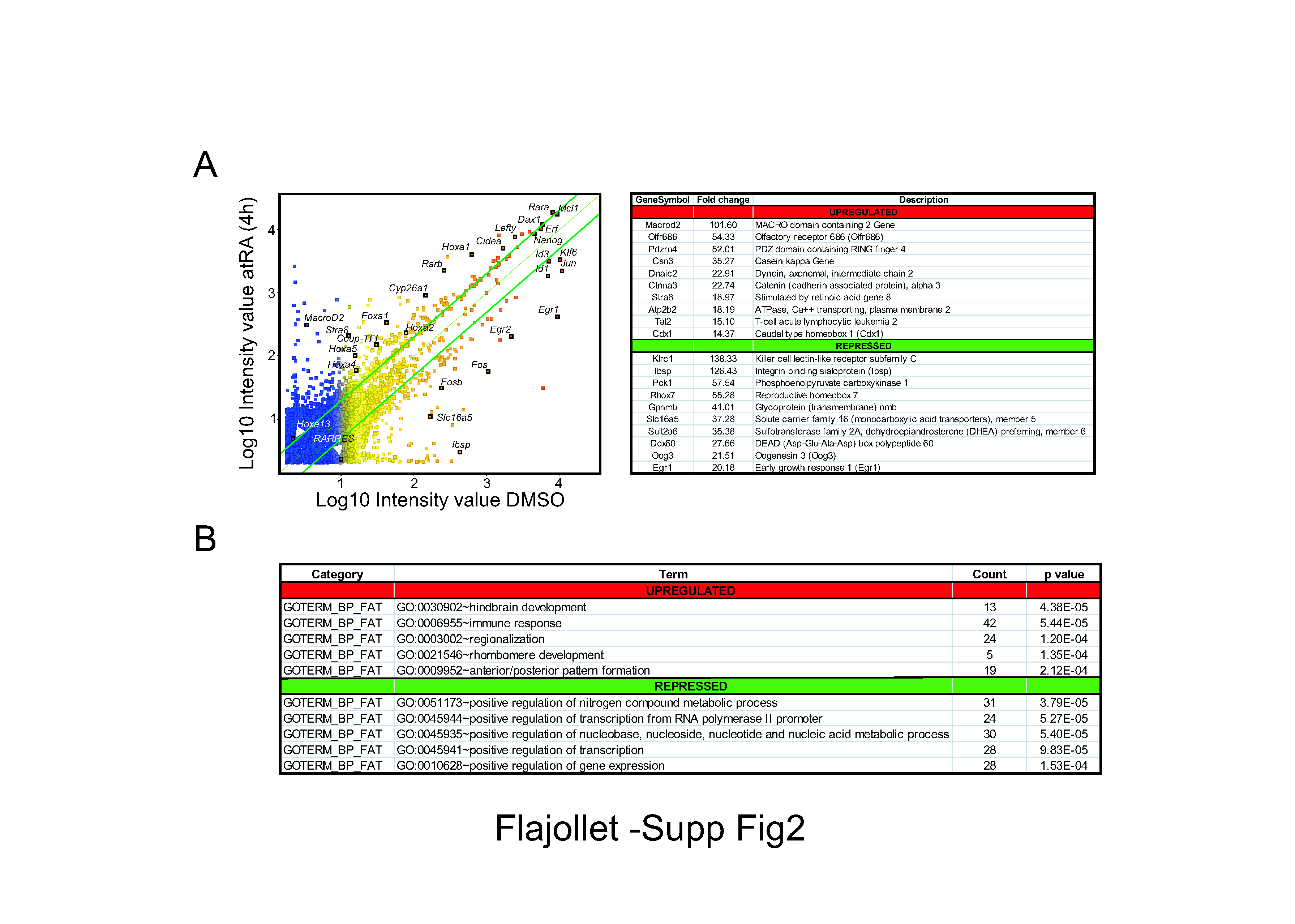

Supplement: Figure S2 — Retinoic acid induces a neuronal differentiation program in EC P19 cells. (A) Microarray gene expression analysis of RA-stimulated P19 cells. A mRNA expression scatter plot was obtained from gene-level interpretation of microarray data (left panel), out of which the 10 most up- or down-regulated genes were identified (right panel). The two thick green lines in the scatter plot indicate a fold change greater than 2. (B) Genes whose expression was modulated more that 2-fold were clustered using the Gene Ontology functional annotation table [1]. (TIFF) [file pone.0064880.s002.tif]

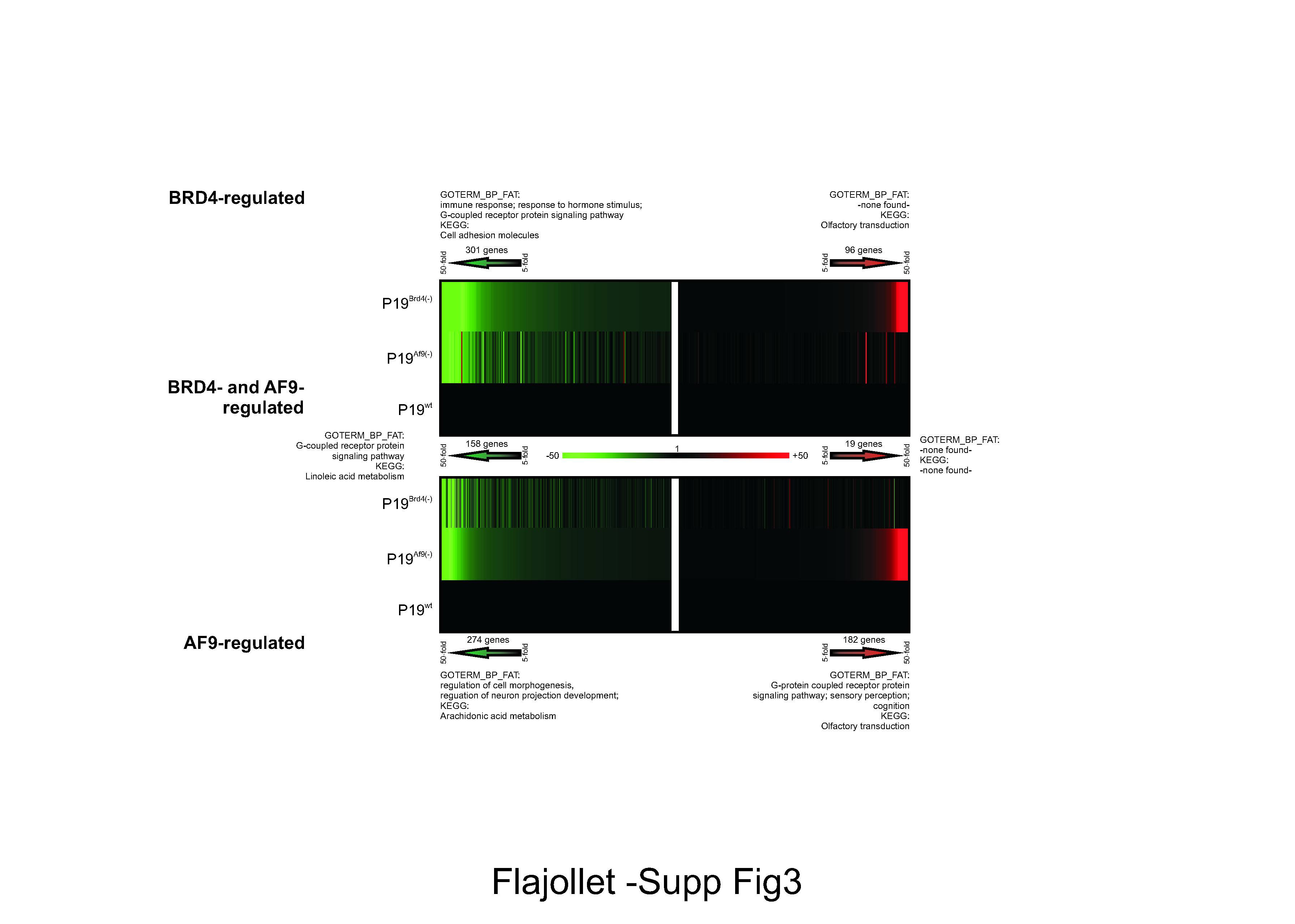

Supplement: Figure S3 — Basal gene expression in Af9- or Brd4-depleted P19 cells. P19wt, P19Brd4(−) or P19Af9(−) cells were treated for 4 hours with 100 nM TTNPB, and mRNAs were extracted and analyzed on Agilent microarrays. Basal expression level was set to 1 in the P19wt background, and genes deregulated by more than 2-fold in both P19Brd4(−) or P19Af9(−) backgrounds were identified using the Genespring software. An entity list consisting of all genes displaying an altered expression in either P19Brd4(−) or P19Af9(−) cells was generated, and their expression level in each background was extracted from microarray data. These expression values, expressed as fold-change over basal in P19wt cells, were processed and used to generate heatmaps with MeV [2]. Genes displaying a FC>5 in the P19Brd4(−) background (top) or in the P19Af9(−) background (bottom) were analyzed using Gene Ontology Functional Annotation Tables and the KEGG database [1]. The most significant terms are indicated. Finally, genes displaying a FC>5 in the P19Brd4(−) background or in the P19Af9(−) background were compared and genes similarly affected by both knockdowns were identified, and subjected to GO FAT and KEGG annotations. These analyses appear in the middle of the figure. Green: down-regulated; red: up-regulated. Raw data are available in Table S3. (TIFF) [file pone.0064880.s003.tiff]

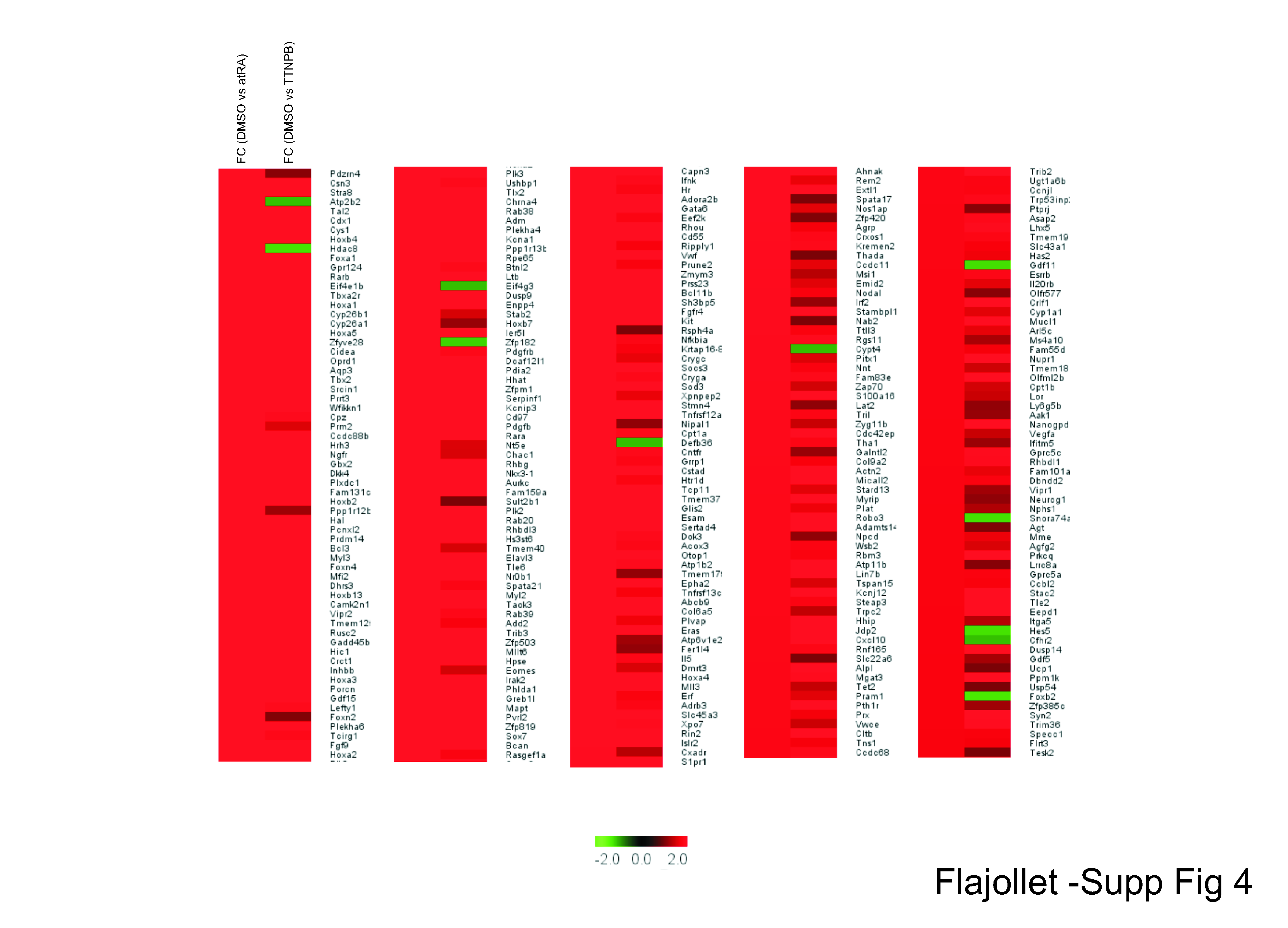

Supplement: Figure S4 — atRA and TTNBP elicits a similar transcriptional program in EC P19 cells. Genes that were modulated with a FC>1.5 at least in one condition (1 µM atRA or 1 µM TTNPB) were identified by data analysis in Genespring v12.0 and the gene list was exported as a text file from this software (available in Table S2). A partial heat map corresponding to upregulated genes (FC>2 in atRA-treated P19) classified by order of induction was generated from this worksheet using MeV and is shown fragmented for visualization purposes from left to right. Up- or downregulated genes (FC>2) are indicated in red or green respectively. (TIFF) [file pone.0064880.s004.tiff]

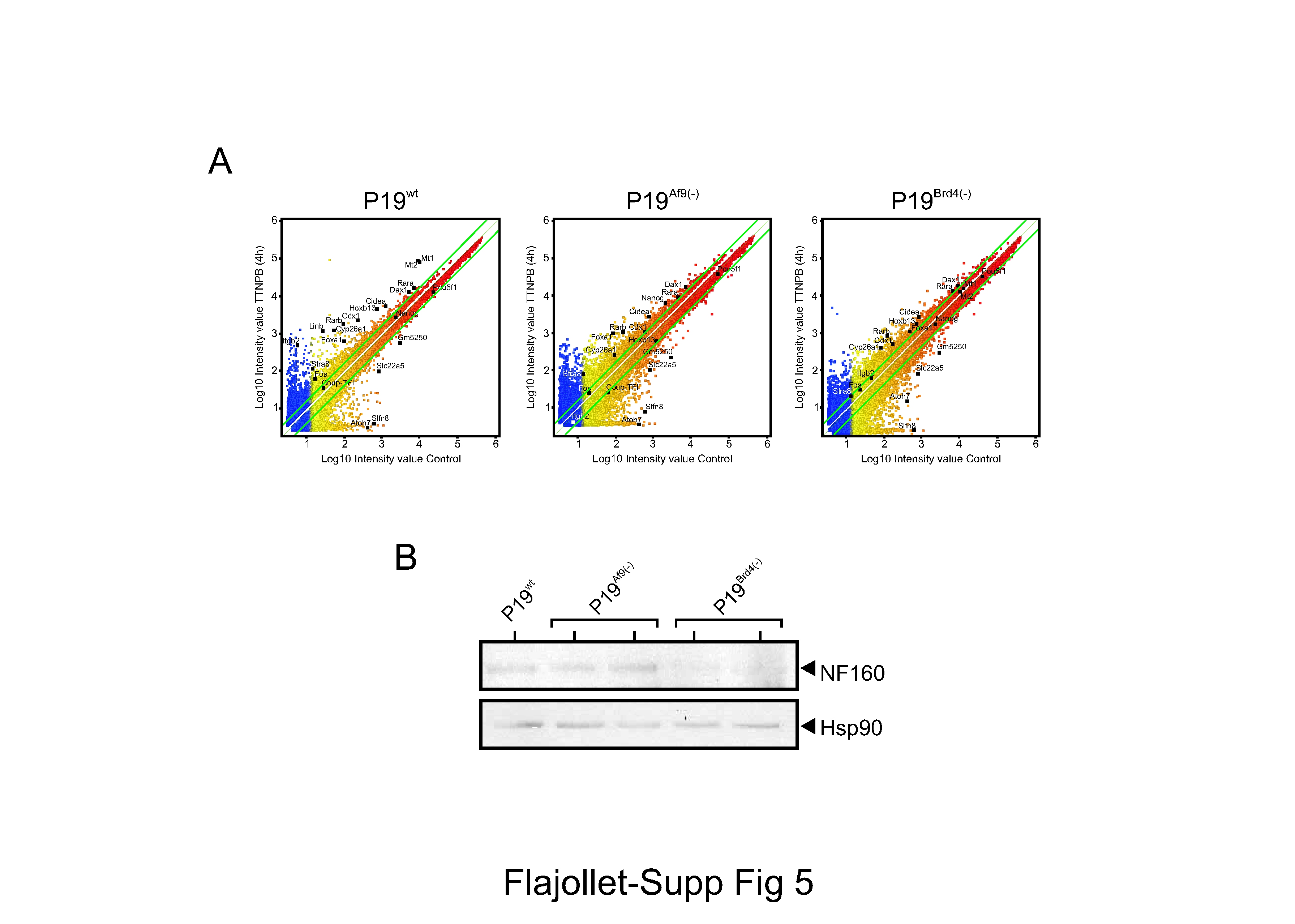

Supplement: Figure S5 — Gene expression induction in an Af9- or Brd4-deficient cellular background. (A) Scatter plot representation of the gene expression pattern in wild type P19 (P19 wt), AF9-depleted P19 or BRD4-depleted P19 (P19Af9(−) or P19Brd4(−) respectively). The two thick green lines in scatter plots indicate a fold change greater than 2. (B) The expression of the neuronal differentiation marker NF160 was assessed by western blot analysis 96 hours after the initiation of neuronal differentiation as previously described [3]. (TIFF) [file pone.0064880.s005.tiff]

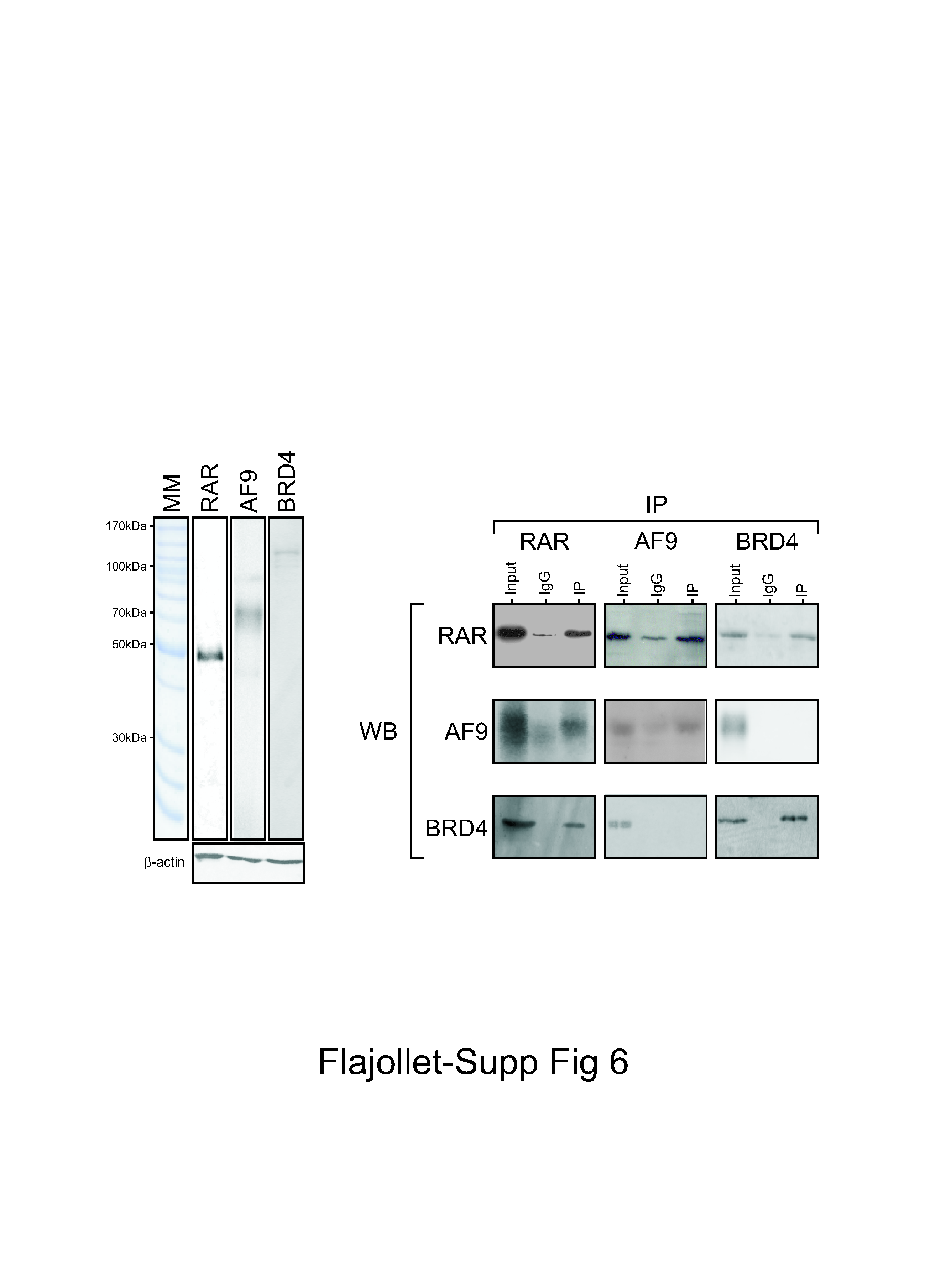

Supplement: Figure S6 — BRD4 and AF9 binds to RAR as distinct complexes. (A) Western blot of P19 whole cell extracts (100 µg proteins). P19 cells were grown under standard conditions and whole cell extracts were prepared as described in the Materials & Methods section. Proteins were resolved on 4–15% polyacrylamide gels and analyzed by western blotting using an anti-RAR (Santa Cruz, sc551), an anti-Brd4 (Active Motif) or an anti-AF9 (Bethyl A300-595A) antibody. (B) Coimmunoprecipitations assays. RAR was immunoprecipitated with an anti-RAR antibody (Santa-Cruz, sc551). Isolated complexes were resolved by 8% SDS-PAGE and analyzed by western-blotting using an anti-Brd4 (Active Motif) or an anti-AF9 antibody (Bethyl A300-595A). Reciprocal immunoprecipitations were carried out using an anti-Brd4 antibody (Santa Cruz H250) or a mix of anti-AF9 antibodies (Bethyl Labs). Input: 10% of total material (500 µg proteins); IgG: non-immune sera (rabbit); IP: immunoprecipitation with the indicated antibody. (TIFF) [file pone.0064880.s006.tiff]
