# Supplementary material for: The Elongation Complex Components BRD4 and MLLT3/AF9 Are Transcriptional Coactivators of Nuclear Retinoid Receptors
Source: PLoS One. 2013 Jun 10;8(6):e64880. doi: 10.1371/journal.pone.0064880 (PMC3677938; doi:10.1371/journal.pone.0064880)
Supplement: Text S1 — References used and cited in the Supporting Information Files. (DOCX) [file pone.0064880.s010.docx]

**REFERENCES**

1. Huang DW, Sherman BT, Lempicki RA (2009) Systematic and integrative analysis of large gene lists using DAVID bioinformatics resources. Nat Protoc 4: 44-57.

2. Saeed AI, Sharov V, White J, Li J, Liang W et al. (2003) TM4: a free, open-source system for microarray data management and analysis. Biotechniques 34: 374-378.

3. Lefebvre B, Brand C, Flajollet S, Lefebvre P (2006) Down-regulation of the tumor suppressor gene retinoic acid receptor β2 through the phosphoinositide 3-kinase/Akt signaling pathway. Mol Endocrinol 20: 2109-2121.

4. Mahony S, Mazzoni EO, McCuine S, Young RA, Wichterle H, et al. (2011) Ligand-dependent dynamics of retinoic acid receptor binding during early neurogenesis. Genome Biol 12: R2.
